# Development and Validation of a Nomogram Based on ^18^F-FDG PET/CT Radiomics to Predict the Overall Survival in Adult Hemophagocytic Lymphohistiocytosis

**DOI:** 10.3389/fmed.2021.792677

**Published:** 2021-12-22

**Authors:** Xu Yang, Jun Liu, Xia Lu, Ying Kan, Wei Wang, Shuxin Zhang, Lei Liu, Hui Zhang, Jixia Li, Jigang Yang

**Affiliations:** ^1^Department of Nuclear Medicine, Beijing Friendship Hospital, Capital Medical University, Beijing, China; ^2^Sinounion Medical Technology (Beijing) Co., Ltd., Beijing, China; ^3^Department of Biomedical Engineering, School of Medicine, Tsinghua University, Beijing, China; ^4^Department of Laboratory Medicine, School of Medicine, Foshan University, Foshan, China; ^5^Department of Molecular Medicine and Pathology, School of Medical Science, The University of Auckland, Auckland, New Zealand

**Keywords:** hemophagocytic lymphohistiocytosis, radiomics, prognosis, ^18^F-FDG PET/CT, nomogram

## Abstract

**Purpose:** Hemophagocytic lymphohistiocytosis (HLH) is a rare and severe disease with a poor prognosis. We aimed to determine if ^18^F-fluorodeoxyglucose (^18^F-FDG) PET/CT-derived radiomic features alone or combination with clinical parameters could predict survival in adult HLH.

**Methods:** This study included 70 adults with HLH (training cohort, *n* = 50; validation cohort, *n* = 20) who underwent pretherapeutic ^18^F-FDG PET/CT scans between August 2016 and June 2020. Radiomic features were extracted from the liver and spleen on CT and PET images. For evaluation of 6-month survival, the features exhibiting *p* < 0.1 in the univariate analysis between non-survivors and survivors were selected. The least absolute shrinkage and selection operator (LASSO) regression analysis was used to develop a radiomics score (Rad-score). A nomogram was built by the multivariate regression analysis to visualize the predictive model for 3-month, 6-month, and 1-year survival, while the performance and usefulness of the model were evaluated by calibration curves, the receiver operating characteristic (ROC) curves, and decision curves.

**Results:** The Rad-score was able to predict 6-month survival in adult HLH, with area under the ROC curves (AUCs) of 0.927 (95% CI: 0.878–0.974) and 0.869 (95% CI: 0.697–1.000) in the training and validation cohorts, respectively. The radiomics nomogram combining the Rad-score with the clinical parameters resulted in better performance for predicting 6-month survival than the clinical model or the Rad-score alone. Moreover, the nomogram displayed superior discrimination, calibration, and clinical usefulness in both the cohorts.

**Conclusion:** The newly developed Rad-score is a powerful predictor for overall survival (OS) in adults with HLH. The nomogram has great potential for predicting 3-month, 6-month, and 1-year survival, which may timely guide personalized treatments for adult HLH.

## Introduction

Hemophagocytic lymphohistiocytosis (HLH) is a syndrome of severe immune activation and dysregulation characterized by hyperactive cytotoxic T lymphocytes, natural killer (NK) cells, and macrophages leading to cytokine storm and immune-mediated multiple organ failure (1, 2). Historically, HLH has been classified as primary or familial HLH driven by underlying genetic defects in cytotoxic immune function or as secondary or reactive HLH caused by infections [e.g., Epstein-Barr virus (EBV), cytomegalovirus (CMV), HIV, and coronavirus disease 2019 (COVID-19)], malignancies (e.g., hematologic malignancies), and autoimmune diseases (e.g., macrophage activation syndrome) (1). Emerging evidence demonstrated that HLH may occur in patients of any age and is most often driven by an integration of genetic defects and acquired exposures (3, 4). Primary HLH occurs in 1/50,000–1/100,000 live-born children, while secondary HLH occurs in older children and adults (1). The precise incidence of adult HLH is still unknown, but it accounts for ~40% of all HLH (1, 5). The frequent manifestations are intermittent fever, hepatosplenomegaly, lymphadenopathy, liver injury, cytopenia, hypertriglyceridemia, hyperferritinemia, and hemophagocytosis (1). Because of few data and/or no prospective studies for adult HLH, pediatric data are often generalized to guide diagnostic, therapeutic, and prognostic decision-making in adults (1, 6). In general, adults have poorer outcome than children even with aggressive therapy, with a median survival of 4 months (1). The principal reasons for mortality are multiorgan failure, hemorrhage, and sepsis, which can be treated properly if diagnosed early (7). Therefore, identifying poor prognosis in adult HLH is crucial for risk stratification and therapeutic decision-making. Recently, it has been reported that clinical and laboratory markers are correlated with survival in adult HLH including age, platelet, fibrinogen, albumin, serum ferritin, alanine aminotransferase (ALT), and malignancy (1, 2, 8, 9). But none of them can be a single effective prognostic factor as a result of poor sensitivity and/or specificity. Hence, it would be of great utility to build a predictive model to precisely evaluate the prognosis in adult HLH based on multiple indictors.

^18^F-fluorodeoxyglucose (^18^F-FDG) PET/CT has been employed for detecting underlying malignancy and predicting prognosis of adult HLH (10–12). One of the most common PET/CT finding is hepatosplenomegaly with diffusely increased FDG uptake, which contains a great deal of information reflecting disease status in adult HLH (13, 14). Radiomics can convert medical images into quantitative data and subsequently analyze these data for prognosis prediction by high-throughput computing. PET/CT radiomic features have been explored to predict outcome in malignancies such as lymphoma and lung cancer (15–17). It has been suggested that the quantitative PET parameters of spleen are independent prognostic factors (11, 12), but whether PET/CT radiomic features extracted from liver and spleen can be applied for outcome prognostication in adult HLH is unclear yet. Therefore, the first aim of this study was to establish a PET/CT radiomics score (Rad-score) for predicting 6-month survival in adult HLH and the second aim was to combine the Rad-score with clinical parameters, in order to develop a nomogram for predicting individual prognosis accurately and reliably.

## Materials and Methods

### Patients

This retrospective study was approved by Institutional Review Board of Beijing Friendship Hospital of Capital Medical University and the requirement of a written informed consent was waived. The medical records of 185 consecutive adult patients (age ≥ 18 years) with a diagnosis of HLH were reviewed from August 2016 to June 2020. The diagnostic criteria of HLH were in accordance with HLH-2004 protocol, which requires five of the following eight criteria: (1) fever; (2) splenomegaly; (3) cytopenia affecting ≥ 2 lineages (Hemoglobin (HGB) < 9 g/dl, platelets < 100 × 10^9^/L, neutrophils < 1.0 × 10^9^/L); (4) serum triglyceride ≥ 265 mg/dl and/or fibrinogen ≤ 150 mg/dl; (5) hemophagocytosis in bone marrow, spleen, lymph nodes, or liver; (6) low or absent NK cell activity; (7) Ferritin ≥ 500 μg/l; and (8) soluble interleukin-2 receptor (soluble CD25) ≥ 2,400 U/ml (18). The exclusion criteria included: patients with receiving chemotherapy before ^18^F-FDG PET/CT scan (*n* = 114) or incomplete follow-up (*n* = 1). Consequently, a total of 70 patients were included in this study. All the patients received personalized treatments in the Department of Hematology and were followed-up for at least 180 days with a median of 353 days. These cases were randomly divided into the training (*n* = 50) and validation cohorts (*n* = 20) with a ratio of 5:2.

### Clinical Data Collection

Clinical parameters including age, gender, malignancy, EBV infection, hemophagocytosis, and laboratory variables [white blood cell, absolute neutrophil, hemoglobin, platelet, C-reactive protein (CRP), ALT, aspartate aminotransferase (AST), triglycerides, serum ferritin, fibrinogen, erythrocyte sedimentation rate, and lactate dehydrogenase] were obtained from medical records (Table 1). All the laboratory and radiological data were collected before initial HLH-specific therapy. The most likely trigger of secondary HLH (malignancy, infection, autoimmune, and idiopathic) was determined by assessment and medical evidence of physician.

**Table 1:** Clinical characteristics of patients in the training and validation cohorts.

**Variables**	**Overall (*n* = 70)**	**Training cohort (*n* = 50)**	**Validation cohort (*n* = 20)**	***p*** **value**
Gender				
Male	36 (51.4%)	23 (46.0%)	13 (65.0%)	0.151
Female	34 (48.6%)	27 (54.0%)	7 (35.0%)	
Age (years)	38 (27–55)	36(24–53)	44(32–60)	0.194
Malignancy				
Yes	22 (31.4%)	15 (30.0%)	7 (35.0%)	0.684
No	48 (68.6%)	35 (70.0%)	13 (65.0%)	
T cell neoplasms				
Yes	11 (15.7%)	8 (16.0%)	3 (15.0%)	0.917
No	59 (84.3%)	42 (84.0%)	17 (85.0%)	
EBV infection				
Positive	34 (48.6%)	23 (46.0%)	11 (55.0%)	0.496
Negative	36 (51.4%)	27 (54.0%)	9 (45.0%)	
Hemophagocytosis				
Yes	48 (68.6%)	34 (68.0%)	14 (70.0%)	0.871
No	22 (31.4%)	16 (32.0%)	6 (30.0%)	
WBC (× 10^9^/L)	4.11 (1.63–6.9)	3.92(1.63–6.02)	5.47 (1.91–10.16)	0.101
ANC (× 10^9^/L)	2.17 (0.94–4.41)	2.13 (0.98–3.82)	2.99 (1.10–6.77)	0.108
HGB (g/L)	92.5 (73.3–108.5)	93 (80–105)	90 (72–111)	0.691
PLT (× 10^9^/L)	85.5 (55.0–179.5)	86 (55–150)	137 (56–194)	0.179
CRP (mg/L)	26 (5–56)	26 (5–49)	34 (11–53)	0.824
ALT (U/L)	53 (32–102)	45 (25–102)	69 (49–96)	0.487
AST (U/L)	68 (33–122)	63 (33–124)	70 (45-118)	0.460
TG (mmol/L)	1.97 (1.43–2.61)	1.79 (1.38–2.47)	2.25 (1.89–3.00)	0.107
SF (ng/ml)	1714.0 (625.6–4075.0)	1450.5 (773.1–3428.5)	3356.5 (1031.3–5359.0)	0.915
FBG (g/L)	2.23 (1.43–3.24)	2.19 (1.42–3.20)	2.63 (1.70–3.13)	0.562
ESR (mm/h)	22 (10–44)	22 (10–38)	24 (12–49)	0.342
LDH (U/L)	547 (343–940)	616 (343–926)	499 (398–764)	0.606

*Data are expressed as median (interquartile range) or number (the proportion of sample size).*

*EBV, Epstein-Barr virus; WBC, white blood cell; ANC, absolute neutrophil count; HGB, hemoglobin; PLT, platelet count; CRP, C-reactive protein; ALT, alanine aminotransferase; AST, aspartate aminotransferase; TGs, triglycerides; SF, serum ferritin; FBG, fibrinogen; ESR, erythrocyte sedimentation rate; LDH, lactate dehydrogenase*.

### ^18^F-Fluorodeoxyglucose PET/CT Imaging Acquisition, Segmentation, and Feature Extraction

^18^F-fluorodeoxyglucose PET/CT was performed on a Siemens biography mCT PET/CT scanner (Siemens Healthineers, Erlangen, Germany). Patients were instructed to fast for at least 6 h, accompanied by blood glucose <11.1 mmol/l. Then, ^18^F-FDG (4.4 MBq/kg) was injected intravenously. After a 60-min uptake time, low-dose CT scan was executed for visualization of anatomic structures and attenuation correction, with 140 keV, automatic mAs, and a slice thickness of 3 mm. The whole-body PET scan was carried out with 2.5 min per bed position using three-dimensional (3D) mode immediately after a whole-body CT scan. Images were reconstructed with an iterative reconstruction algorithm.

**Figure 1:**
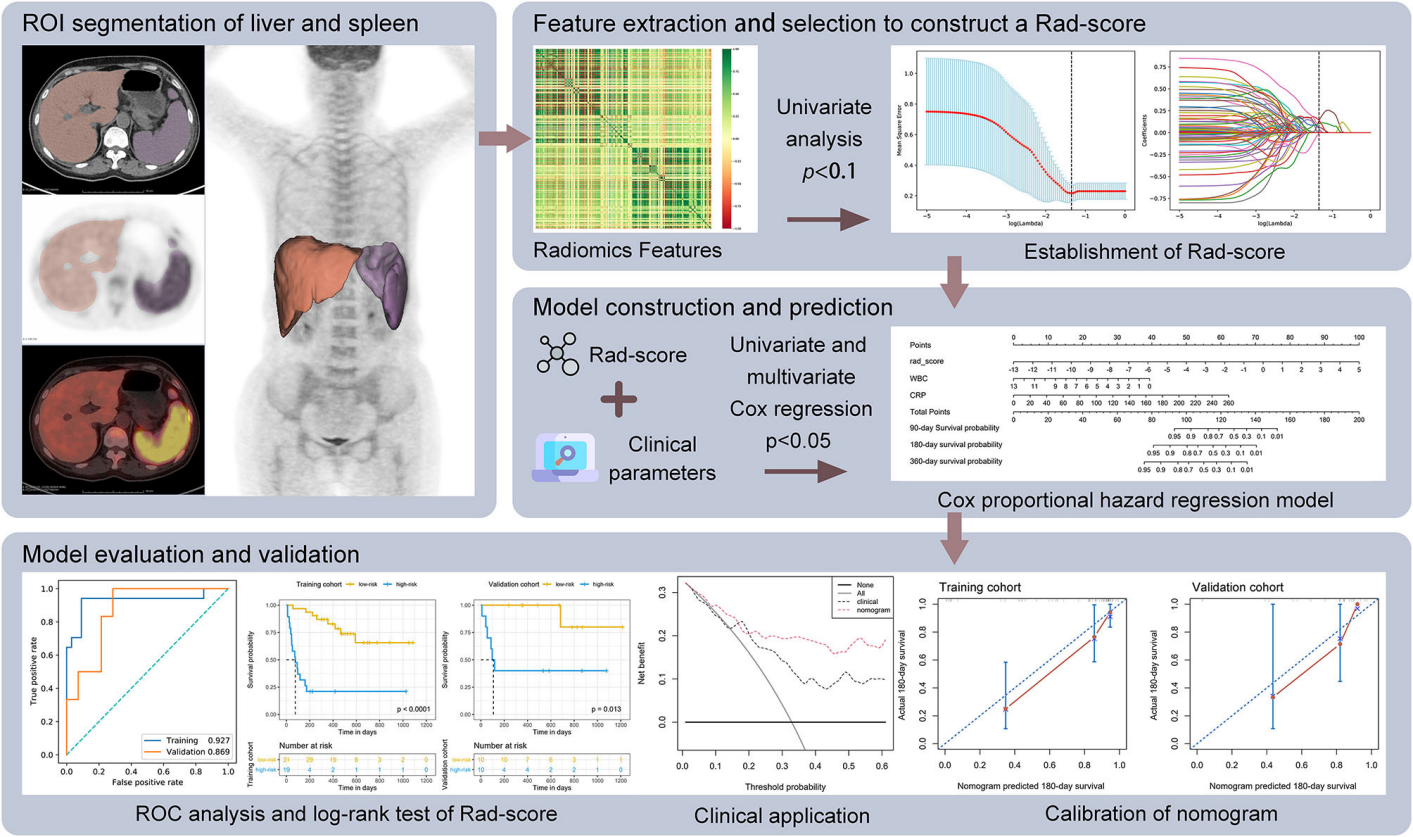
Workflow of radiomics analysis.

The entire liver and spleen on CT images were defined as the regions of interest (ROIs), which were delineated by two experienced nuclear radiologists with a validated semi-automatic approach using (3D Slicer^*TM*^ software, Boston, Massachusetts, United States) (version 4.10.0, http://www.slicer.org) (Figure 1) (19). Moreover, the ROIs were resampled exploiting B-spline interpolation in order for mapping those onto the PET images. In consequence, the ROIs had the matching pixel spacing with the PET images.

### Radiomic Feature Extraction

Radiomic features were extracted from ^18^F-FDG PET and CT images separately, using pyradiomics that is an open-source Python package (20). These included first-order features (*n* = 18), shape features (*n* = 14), gray level co-occurrence matrix (GLCM) features (*n* = 24), gray level run length matrix (GLRLM) features (*n* = 16), gray level size zone matrix (GLSZM) features (*n* = 16), neighboring gray tone difference matrix (NGTDM) features (*n* = 5), and gray level dependence matrix (GLDM) features (*n* = 14). Image processing utilized wavelet filtering, square, square root, logarithm, exponential, and gradient. Overall, a total of 5,264 radiomic features (4 × 1,317) were obtained from liver and spleen on PET and CT images.

### Radiomic Feature Selection and the Rad-Score Construction

Our workflow is shown in Figure 1. Firstly, the univariate analysis (*t*-test for normally distributed variables or the Mann–Whitney *U* test for skewed distributed variables) was used to compare differences of radiomic features between non-survivors and survivors at 180 days in the training set. The total of 384 features with *p*-values < 0.1 were retained for further analysis. Next, the least absolute shrinkage and selection operator (LASSO) algorithm was applied to select the optimal features among 384 features in the training set, adding L1 regularization term to a least square algorithm for data dimension reduction. Because of imbalanced datasets, the synthetic minority oversampling technique (SMOTE) was used to improve random oversampling in the training set. An individualized Rad-score was calculated from a linear combination of the selected features weighted by their respective coefficients. The receiver operating characteristic (ROC) curve was employed to evaluate the prediction accuracy and determine the optimal threshold of the Rad-score. All the patients were divided into high- and low-risk groups according to the maximum Youden index of the ROC curve. The potential association of the Rad-score with overall survival (OS) was evaluated by the Kaplan–Meier survival analysis and the log-rank test in the training and validation cohorts.

### Clinical Variables Selection and Nomogram Creation

To build a powerful model and a robust nomogram for the survival prediction, the clinical prognostic factors were chosen by the univariate Cox regression analyses (*p* < 0.05). Then, the Rad-score and the strong clinical indicators were incorporated to establish the multivariate Cox regression model that was visualized by a nomogram. The Harrell's concordance-index (C-index) was employed to assess the model performance and calibration curves were plotted to enhance the predictive precision of nomogram. Similarly, a clinical model was established with clinical information alone by the multivariate Cox regression analysis. Three different types of predictive models (clinical variables, the Rad-score, and their combinations) were evaluated by the C-index. Decision curve analysis (DCA) was utilized to assess the clinical usefulness of the models.

### Statistical Analyses

Continuous variables are presented as medians with interquartile ranges and categorical variables are presented as frequencies and percentages. OS was defined as the time from the initial diagnosis of HLH to the date of death from any cause or deadline of follow-up. All the *p*-values were two-sided, with a significant level of <0.05. Statistical analyses were performed with Python (version 3.7.8, www.python.org) and R (version 4.0.3, www.r-project.org). The Python packages “sklearn,” “numpy,” and “pandas” were used for the LASSO binary logistic regression and the ROC curve; the “scipy” was for analyzing statistical properties; and the “imblearn” was for analyzing SMOTE. The R package “rms” was employed to create nomograms.

## Results

### Baseline Clinical Characteristics of Patients

A total of 70 adults with HLH were included in this study who fulfilled the inclusion criteria. There were 36 males and 34 females and the median age at diagnosis was 38 years (range: 18–79 years). The baseline characteristics of all the patients are shown in Table 1.

The possible triggers of HLH in these patients were as follows: 35 (50.0%) infections, 22 (31.4%) malignancies, 7 (10%) autoimmune diseases, and 6 (8.6%) unknown disorders. In the 35 cases with infectious disorders, viral infections were the most common cause with 23 (65.7%) EBV, 3 (8.6%) CMV, and 5 (14.3%) other viruses. Bacterial infections were identified in 4 (11.4%) patients. Among 22 malignancy-associated HLH cases, diffuse large B-cell lymphoma (*n* = 6, 27.3%) and NK/T-cell lymphoma (*n* = 6, 27.3%) were the most frequent triggers. The other malignancy-associated patients with HLH were two classical Hodgkin's lymphoma, two peripheral T-cell lymphoma, two unclassified T-cell lymphoma, one follicular lymphoma, one non-Hodgkin B cell lymphoma, one anaplastic large cell lymphoma, and one acute lymphocytic leukemia. Concomitant malignancies and EBV infection were found in 11 (15.7%) patients. Adult Still's disease (*n* = 5, 71.4%) was the most common diagnosis among autoimmune diseases and the other two patients were diagnosed with systemic lupus erythematosus and undifferentiated systemic rheumatic disease, respectively.

The baseline characteristics in the training and validation cohorts are also given in Table 1. Obviously, the clinical variables had no differences between the two cohorts (*p* > 0.05). After a median follow-up of 353 days (range: 9–1,216 days), 30 patients (42.8%) had died.

### Radiomic Feature Selection and the Rad-Score Construction and Evaluation

**Figure 2:**
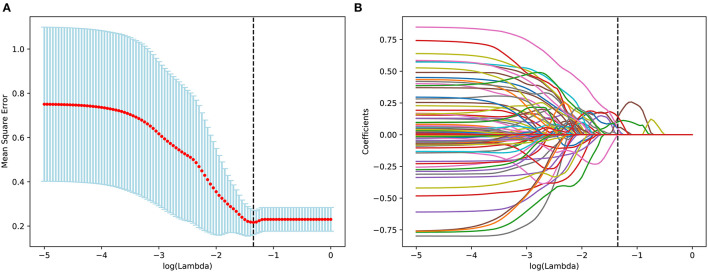
Feature selection for the prediction using the least absolute shrinkage and selection operator (LASSO) regression model, tuning parameter (λ) selection in the LASSO model involved the use of tenfold cross-validation **(A)**. In the coefficient profiles of the radiomics features for OS prediction, a value of Lambda = 0.044668 was selected as the optimal value **(B)**.

The optimal radiomic features were selected by the LASSO algorithm and 10-fold cross-validation (Figure 2). Eventually, six features were extracted to construct the Rad-score in the training set, which included 3 CT features and 3 PET features. The selected features were spleen_CT_wavelet-HHH_GLSZM_gray level non-uniformity normalized, spleen_CT_wavelet-LHL_NGTDM_contrast, liver_CT_wavelet-HHH_GLCM_informational measure of correlation (IMC) 2, spleen_PET_square root_first order_kurtosis, spleen_PET_wavelet-LHL_GLSZM_size zone non-uniformity normalized, and liver_PET_wavelet-HHL_GLSZM_small area emphasis. Among these features, there were four from spleen and two were from liver. The Rad-score for each patient was calculated by the following formula:

Rad-score = 13.762 + 300.60 × spleen_CT_wavelet-LHL_NGTDM_contrast – 9.1753 × spleen_CT_wavelet-HHH_GLSZM_gray level non-uniformity normalized – 0.17938 × spleen_PET_square root_first order_kurtosis + 13.305 × spleen_PET_wavelet-LHL_GLSZM_size zone non-uniformity normalized – 246.77 × liver_CT_wavelet-HHH_GLCM_IMC 2 + 4.2599 × liver_PET_wavelet-HHL_GLSZM_small area emphasis.

**Figure 3:**
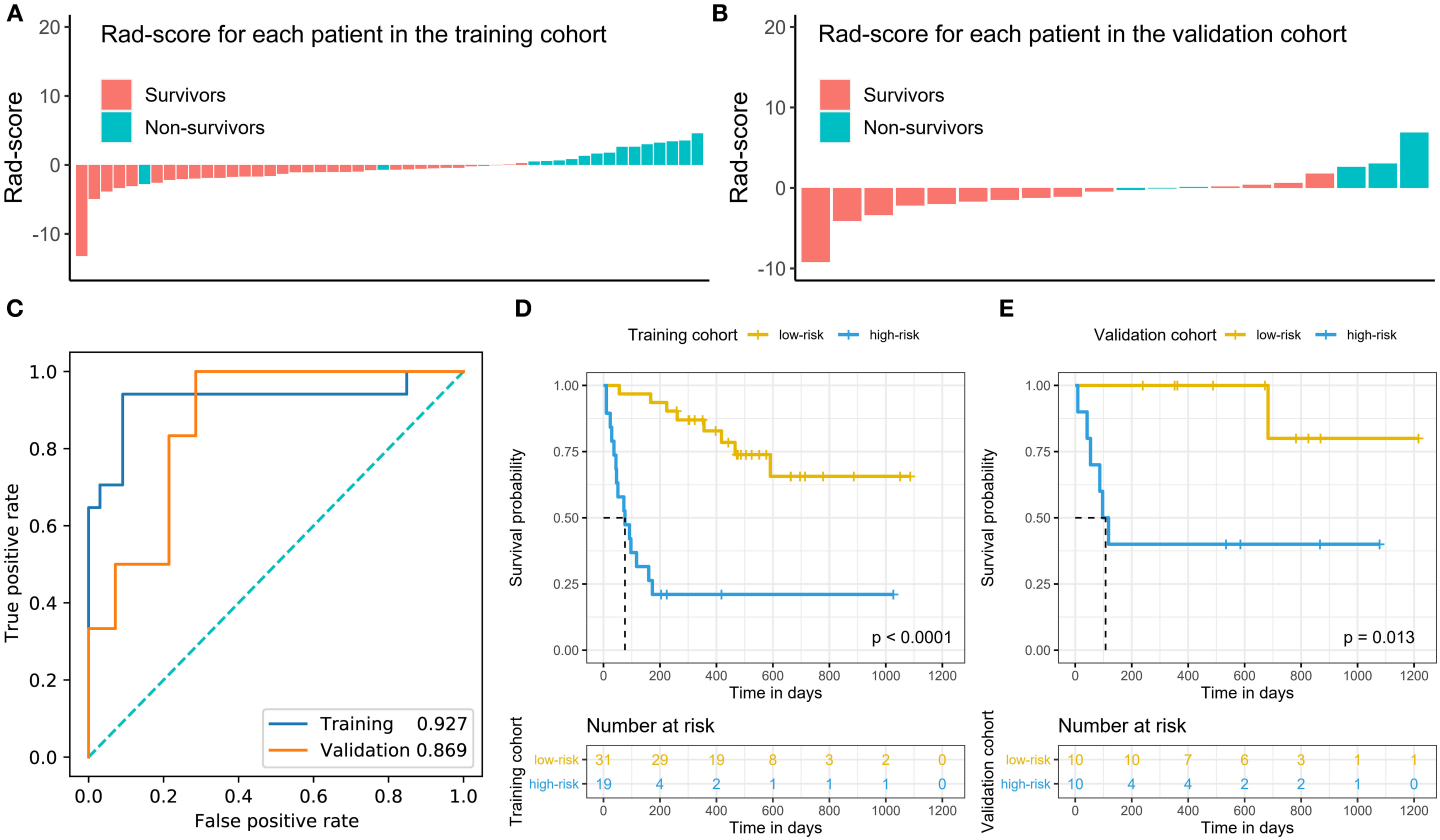
Rad-score of patients in the training and validation cohorts **(A,B)** and time-dependent ROC analysis of Rad-score at 180 days **(C)**. Kaplan-Meier survival analysis with the best cutoff value of the Rad-score in the training cohort **(D)** and validation cohort **(E)**. We calculated *p* values using the log-rank test.

The median and the interquartile range for the selected radiomics features in the training cohort are shown in Table 2. The Rad-score in the training and validation cohorts are shown in Table 3. Not surprisingly, the Rad-score had notable difference between non-survivors and survivors in the training (*p* < 0.001) and validation cohorts (*p* = 0.011). Particularly, non-survivors had the higher Rad-score than survivors in the training (Rad-score = 1.6386 vs. −1.0608) and validation cohorts (Rad-score = 1.3763 vs. −1.3595). The Rad-score for individuals in the both the cohorts is shown in Figures 3A,B.

**Table 2:** Comparison of the radiomics features between 180-day survivors and non-survivors in the training cohort.

**Radiomic features**	**Survivors (*n* = 33)**	**Non-survivors (*n* = 17)**	***p*** **value**
spleen_CT_wavelet-LHL_ngtdm_Contrast	0.0024 (0.0016–0.0045)	0.0037 (0.0020–0.0050)	0.072
spleen_CT_wavelet-HHH_glszm_Gray Level Non-Uniformity Normalized	0.4259 (0.3884–0.5000)	0.4102 (0.3333–0.4404)	0.045
spleen_PET_squareroot_firstorder_Kurtosis	4.8251 (3.9777–6.1500)	5.7335 (4.1330–7.8258)	0.035
spleen_PET_wavelet-LHL_glszm_Size Zone Non-Uniformity Normalized	0.1927 (0.1641–0.2355)	0.2388 (0.2000–0.2800)	0.004
liver_CT_wavelet-HHH_glcm_Imc2	0.0712 (0.0682–0.0725)	0.4500 (0.4222–0.5185)	0.082
liver_PET_wavelet-HHL_glszm_Small Area Emphasis	0.3890 (0.2778–0.5284)	0.3890 (0.2778–0.5284)	0.084

*Data are expressed as median (interquartile range)*.

**Table 3:** Comparison of the Rad-score between 180-day survivors and non-survivors in both the training and validation cohorts.

	**Training cohort (*n* = 50)**		***p*** **value**
	**Survivors (*n* = 33)**	**Non-survivors (*n* = 17)**	
Rad-score	−1.0608 (−1.9723–−0.6384)	1.6386 (0.5678–2.9977)	<0.001
	**Validation cohort (*n* = 20)**		***p*** **value**
	**Survivors (*n* = 14)**	**Non-survivors (*n* = 6)**	
Rad-score	−1.3595 (−2.1365–0.0290)	1.3763 (−0.0232–2.9420)	0.011

*Data are expressed as median (interquartile range)*.

In addition, the Rad-score had good predictive power for survival forecast at 180 days and its area under the ROC curves (AUCs) in distinguish high-risk status were 0.927 (95% CI: 0.879–0.975) in the training set and 0.869 (95% CI: 0.684–1.000) in the validation set (Figure 3C). The best cutoff with maximum Youden index was −0.3 and, therefore, patients were divided into high- and low-risk groups according to the Rad-score in the both the cohorts. The Kaplan–Meier curves and the log-rank test found that patients in low-risk category had a better prognosis than those in high-risk category in the training and validation cohorts (*p* < 0.05) (Figures 3D,E).

### Strong Predictor Selection and Model Establishment and Assessment

The univariate Cox regression analysis showed that 6 parameters were significantly associated with OS including the Rad-score, T-cell neoplasms, white blood cell, hemoglobin, platelet count, and CRP (*p* < 0.05; Table 4). The multivariate analysis displayed that the Rad-score, white blood cell, and CRP were consistently strong predictors (Table 4), which were used to build the combined model. When the Rad-score was excluded, three variables (T-cell neoplasms, hemoglobin, and platelet count) were independent prognostic factors among clinical parameters (Table 5). Likewise, these three prognostic factors were used to build the clinical model.

**Table 4:** The univariate and multivariate Cox hazards regression analysis of OS in the training cohort.

**Variables**	**Univariate analysis**		**Multivariate analysis**	
	**Hazard ratio (95% CI)**	***p*** **value**	**Hazard ratio (95% CI)**	***p*** **value**
Rad-score	1.683 (1.312–2.158)	<0.001	1.522 (1.221–1.896)	<0.001
Gender	1.930 (0.844–4.412)	0.119		
Age	1.017 (0.992–1.044)	0.185		
Malignancy	1.569 (0.676–3.640)	0.295		
T cell neoplasms	3.304 (1.292–8.448)	0.013		
EBV infection	2.240 (0.966–5.195)	0.060		
Hemophagocytosis	1.176 (0.483–2.861)	0.721		
WBC	0.790 (0.661–0.945)	0.010	0.796 (0.661–0.957)	0.015
ANC	0.825 (0.656–1.039)	0.102		
HGB	0.971 (0.951–0.992)	0.006		
PLT	0.991 (0.984–0.997)	0.007		
CRP	1.007 (1.002–1.013)	0.009	1.018 (1.010–1.027)	<0.001
ALT	0.996 (0.990–1.002)	0.230		
AST	0.999 (0.997–1.001)	0.518		
TG	0.827 (0.500–1.366)	0.458		
SF	1.000 (1.000–1.000)	0.202		
FBG	0.900 (0.662–1.224)	0.502		
ESR	0.997 (0.981–1.013)	0.702		
LDH	1.000 (0.999–1.001)	0.756		

*OS, overall survival; EBV, Epstein-Barr virus; WBC, white blood cell; ANC, absolute neutrophil count; HGB, hemoglobin; PLT, platelet count; CRP, C-reactive protein; ALT, alanine aminotransferase; AST, aspartate aminotransferase; TGs, triglycerides; SF, serum ferritin; FBG, fibrinogen; ESR, erythrocyte sedimentation rate; LDH, lactate dehydrogenase*.

**Table 5:** The multivariate Cox hazards regression analysis of OS in the training cohort without the Rad-score.

**Variables**	**Hazard ratio (95% CI)**	***p*** **value**
T cell neoplasms	5.800 (1.957–17.187)	0.002
HGB	0.999 (0.946–0.986)	<0.001
PLT	0.994 (0.987–1.000)	0.050

*HGB, hemoglobin; PLT, platelet count*.

**Figure 4:**
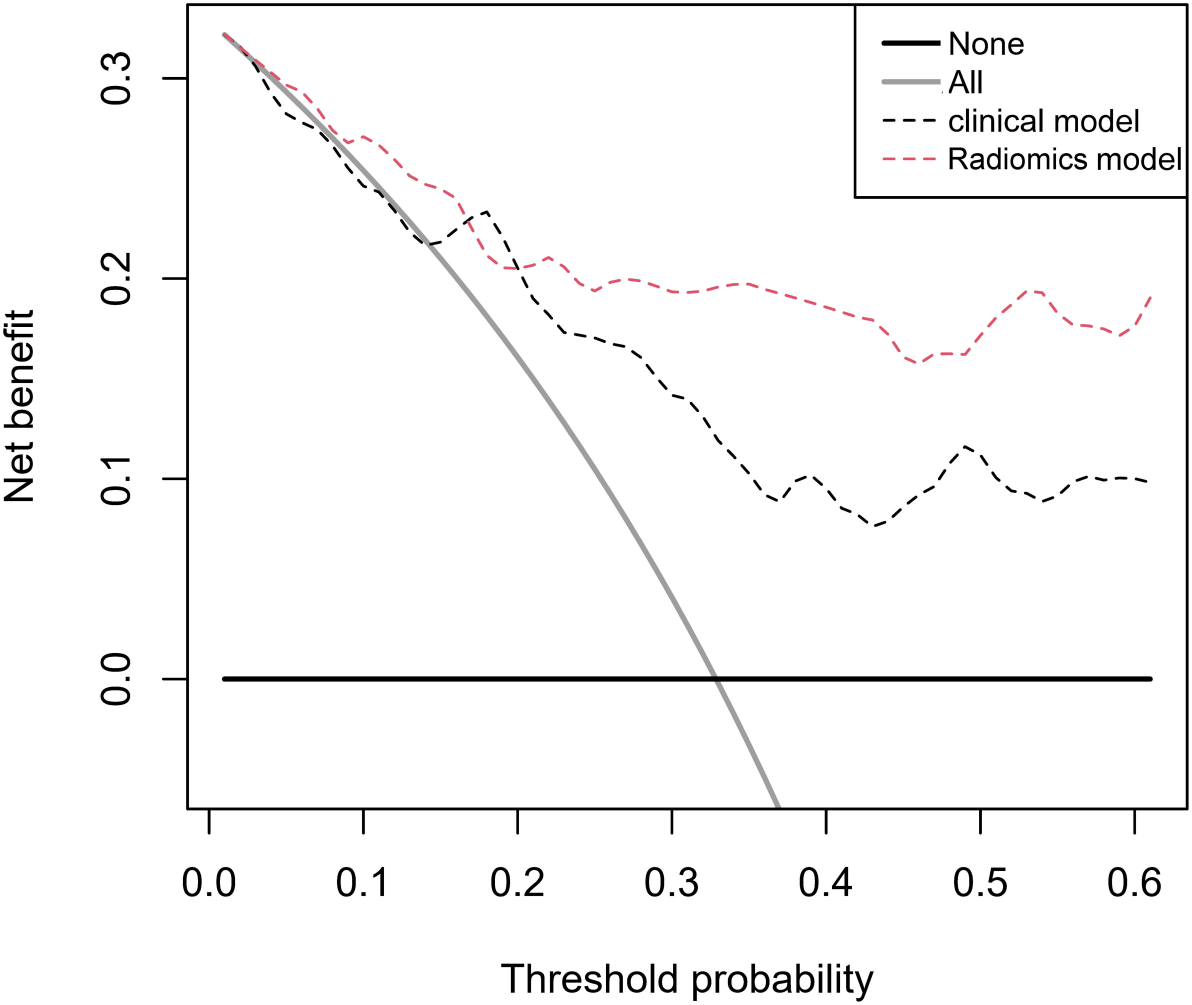
Decision-curve analysis for the radiomics model and clinical model. The threshold probability represents the predicted 180-day risk of death for recommending aggressive treatment.

To assess the performance of models in predicting prognosis, the C-indices of three types of models were shown in Table 6. The Rad-score model had acceptable predictive ability with C-indices of 0.795 (95% CI: 0.695–0.895) and 0.752 (95% CI: 0.591–0.913) in the training and validation cohorts, respectively. The C-indices of the clinical model were of 0.765 (95% CI: 0.665–0.865) and 0.762 (0.527–0.997) in the training and validation cohorts, respectively. It was noticeable that the combined radiomics model had the highest C-indices, with 0.831 (95% CI: 0.749–0.913) and 0.810 (95% CI: 0.657–0.963) in both the training and validation cohorts, sequentially. The curves of decision-curve analysis (DCAs) indicated that the combined radiomics model provided more net clinical benefit than clinical model with a threshold > 0.25 (Figure 4).

**Table 6:** Model performance.

**Model**	**Training cohort**		**Validation cohort**	
	**C-index**	**95% CI**	**C-index**	**95% CI**
Rad-score	0.795	0.695–0.895	0.752	0.591–0.913
Clinical model	0.765	0.665–0.865	0.762	0.527–0.997
Combined radiomics model	0.831	0.749–0.913	0.810	0.657–0.963

*C-index: Harrell's concordance-index*.

### Personalized Nomogram Establishment and Validation

**Figure 5:**
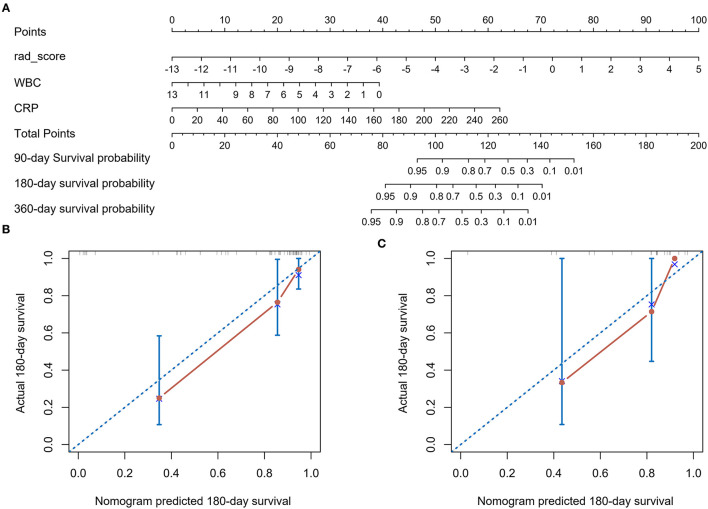
The radiomics nomogram for predicting overall survival for adult HLH patients **(A)**. Points for Rad-score, WBC, and CRP can be obtained by calibrating with the point caliper, and then combined to obtain a total score that can be calibrated with the patient's probability of survival at different time. Calibration curves of the radiomics nomogram in the training cohort **(B)** and validation cohort **(C)**. Nomogram-estimated OS is plotted on the x-axis; the observed OS is plotted on the y-axis. The diagonal dotted line is a perfect estimation by an ideal model, in which the estimated outcome perfectly corresponds to the actual outcome. The solid red line represents performance of the nomogram: A smaller distance of the scatter points from the dotted line indicates better calibration.

Given that the combined model possessed synergetic power for survival prediction, the personalized nomogram was constructed by incorporating all the three independent prognostic factors (the Rad-score, white blood cell, and CRP) (Figure 5A), which can visualize the prediction outcome and the proportion of each factor. The calibration curves demonstrated good agreements between the predicted and observed values in the training and validation cohorts, indicating that the nomogram was able to precisely predict 6-month survival (Figures 5B,C).

## Discussion

Timely diagnosis and prognosis are critical for HLH considering that the early and proper administration of an efficacious therapy can improve survival. In this study, the novel prognostic factors and predictive models associated with 6-month survival in adult patients with HLH are reported *via* pretherapeutic ^18^F-FDG PET/CT radiomics analysis. The Rad-score and the combined prediction model (the Rad-score and clinical variable combination) have been developed for quantitative identification of the adults with HLH at high risk of death within 6 months in 70 patients.

^18^F-fluorodeoxyglucose PET/CT is a whole-body scan containing both the metabolic and anatomical information, which has been recommended for identifying possible triggers and suitable biopsy sites in secondary HLH (6). However, ^18^F-FDG PET/CT findings are non-specific, since inflammatory response and malignant lesions have the same manifestation that is hypermetabolism. In HLH, ^18^F-FDG PET/CT often shows diffusely increased FDG uptake in spleen, liver, and bone marrow with or without focal lesions and hypermetabolic lymph nodes. Increasing evidence demonstrated that these non-specific presentations can be used to assess systemic inflammatory response and have potential for prognosis prediction in HLH (21). For instance, the FDG uptake of spleen and bone marrow has been considered as prognostic factors in adult patients with HLH (11, 12, 22). More importantly, spleen and liver, components of the reticuloendothelial system, are the most frequent abnormal signs in HLH (23). Our data proved that 6 radiomic features from spleen and liver were linked with the prognosis of adult HLH, thus utilized for establishment of the Rad-score. Among six radiomic features, two-thirds (4/6) were derived from spleen including GLSZM size zone non-uniformity normalized feature and kurtosis of spleen PET and GLSZM gray level non-uniformity normalized feature and NGTDM contrast of spleen CT. It is well-known that spleen is the largest secondary lymphoid organ and a site where immune responses can be controlled by activated immune cells. As splenomegaly is one of the diagnostic criteria of HLH, the hypermetabolic spleen has been discovered to be correlated with high inflammatory response and cytokine activity (24, 25). One recent report suggested that spleen FDG uptake may provide useful information for predicting in-hospital mortality in autoimmune diseases including HLH (26). Another study of 43 patients with secondary HLH found that the ratio of spleen to mediastinum in the average standardized uptake value (SUV) was an independent predictor for survival (12). In consistent with these statements, our findings indicated that radiomic features of spleen possessed a powerful predictive ability for 6-month survival in adult patients with HLH. The rest of two radiomic features were extracted from liver including GLCM IMC2 (Informational Measure of Correlation) of liver CT and GLSZM small area emphasis of liver PET. Radiomics have showed great value in characterization of diffuse liver diseases such as non-alcoholic steatohepatitis and chronic hepatitis B (27). In addition, the well-known liver enzymes, AST and ALT, are identified as indicators of various diseases including HLH. High ALT and AST/ALT ratio have been found to act as adverse prognostic factors in adult HLH (8, 28). Furthermore, hepatic involvement and hepatomegaly reveal poor prognostic indicators and early death predictors in HLH (8, 28). In line with these studies, our results illustrated that the radiomic features of spleen and liver presented great prognostic values for adult HLH.

Radiomics is a high-throughput extraction of quantitative information from medical images as well as subtle manifestations that are difficult to recognize or quantify by human eyes. Compared with the traditional PET/CT metrics, the radiomic features may reflect the pathological process much more sufficiently in the spleen and liver of patients with HLH. In this study, the majority of the selected radiomic features (5/6) were derived from wavelet decomposition images, indicating that wavelet transforms emphasize image details. It is very likely that wavelet decomposition images contain inconspicuous prognostic information (29, 30). GLSZM quantifies the number of groups of interconnected neighboring voxels with the same gray level intensity. NGTDM represents contrast, quantifying the difference between the gray level of a voxel and the average level of its neighbors within a distance. GLCM captures spatial relationships of pairs of voxels, while kurtosis is a first-order feature expressing the peak of the distribution of values in the ROI. All the selected features describe the texture of the spleen and liver quantitatively, reflecting uniformity or heterogeneity in both the organs (31). Previous studies found that intratumor heterogeneity was associated with poor outcome in various malignancies (15, 17, 30, 32, 33). HLH is a heterogeneous disease with various etiological components and complex underlying genetic variant types (3). Each possible etiology has distinct clinical characteristics and prognosis. Even in lymphoma-associated HLH that has the worst prognosis, the treatment response is diverse (34). HLH could occur in EBV-associated T-/NK-cell lymphoproliferative disorders, which is a spectrum of disease from infection to malignancy. The histological features and immunophenotype are markedly heterogeneous. As in children, multiple gene mutations are linked to the development of HLH in adults, especially with the EBV-driven lymphoma, which requires hematopoietic cell transplant (6, 35, 36). The ^18^F-FDG appearance of the liver and spleen came in various sizes, densities, and metabolisms, which reflected the heterogeneity of HLH. Radiomics quantified the spatial complexity of them. This study exhibited that the heterogeneity of spleen and liver may reveal overproliferation of immune cells accompanied with inflammatory infiltration triggered by EBV infection (37–40). On the other hand, the heterogeneous distribution of metabolism or density may also suggest the involvement of tumor cells (41–43). Both the malignancy and EBV infection seemed to link an inferior prognosis in adult HLH (9, 34, 44, 45). Additionally, the radiomic features have the possibilities associated with genetic signatures (3, 46); however, the underlying biological significance of these radiomic features has not been fully studied and the relationships among radiomic features, genetic signatures, and prognosis need further exploration in HLH.

Recent studies pointed out that a number of clinical parameters play a role in the prognosis of HLH such as lymphoid malignancy, hemoglobin, platelets, CRP, and cytopenia [(8, 9, 50, 51)]. It is well-documented that lymphoid malignancy is negatively associated with survival. Typically, T-cell lymphoma is acknowledged to have a more severe survival due to poor response to chemotherapy, in comparison with B-cell lymphoma (47). In a large-scale Japanese study, the 5-year OS was the worst in T-/NK-cell lymphoma-associated HLH compared with other types of HLH including primary HLH, B-cell lymphoma-associated HLH, and infection-associated HLH (48). Lower hemoglobin and platelet have been reported to be the more consistent negative prognostic biomarkers in HLH (8, 49). This study consistently showed that these 3 clinical parameters were involved in the clinical prediction model. However, the two clinical variables incorporated in the nomogram were white blood cell and CRP. Cytopenia is one of the major presentation in HLH. Serious cytopenia may mark the severity of a cytokine storm and lead to hemorrhage and sepsis, suggesting to be an inferior factor (7). CRP is the prototypical acute phase serum protein, increasing rapidly during inflammation (50). It has been highlighted that CRP is markedly enhanced in patients with secondary HLH compared to primary ones. High CRP levels have been correlated with increased risk of infection and overall mortality in HLH, suggested to be indices of disease severity (51). CRP probably serves as a predictor of ^18^F-FDG PET/CT effectiveness due to the fact that the diagnostic accuracy of PET/CT is positively linked with CRP > 60 mg/l in HLH (12).

Interestingly, T-cell neoplasms were not retained in the predictive model when the Rad-score was incorporated. A possible explanation was that the Rad-score contained partial pathological information. The inclusion of the Rad-score not only improved the prognostic performance, but also simplified the prediction model. DCA demonstrated that the nomogram with the Rad-score and two clinical parameters was superior to the clinical model in terms of clinical application. Overall, the nomogram was successfully built to predict 3-month, 6-month, and 1-year survival of adults with HLH and the accuracy and clinical applicability of the model were verified through C-index, calibration curve, and DCA.

This study has several limitations. First, patients may have been missed for inclusion in a single-center study and selection bias may occur because of the retrospective nature of the study design. Second, the heterogeneity of the patients and treatments may affect our results. Third, gene, transcript, and protein signatures become increasingly important for the prognosis of adult HLH (3), but these data were not collected. Finally, the Rad-score was calculated using ROIs that were manually delineated in 3D slicer. It was time-consuming and inconvenient for clinical practice, so automatic or semi-automatic image segmentation will be needed. Notably, a multicenter and prospective study with larger cohort will be required to validate our findings in the future.

## Conclusion

This preliminary study indicated that the pretherapeutic ^18^F-FDG PET/CT radiomic features of spleen and liver are independent prognostic factors in adult HLH, with the heterogeneity of spleen and liver associated with inferior prognosis. Integrating radiomic features with clinical parameters show synergetic power for 6-month survival prediction compared to other models with radiomics features or clinical parameters alone. The nomogram has great potential for predicting individualized 3-month, 6-month, and 1-year survival, which may timely guide personalized treatments for adult HLH.

## Data Availability Statement

The raw data supporting the conclusions of this article will be made available by the authors, without undue reservation.

## Ethics Statement

The studies involving human participants were reviewed and approved by Institutional Review Board of Beijing Friendship Hospital of Capital Medical University. Written informed consent for participation was not required for this study in accordance with the national legislation and the institutional requirements. No potentially identifiable human images or data are presented in the manuscript.

## Author Contributions

JY, YK, and HZ contributed to the study design, decision-making, and coordination of the study. XY, JLiu, XL, WW, and SZ contributed to the management of registration of cases and collected PET/CT image data. XY, JLiu, XL, WW, and YK contributed to the image quality control, analysis, and data interpretation. LL and HZ contributed to the statistical analysis. XY, JLi, and JY contributed to the drafting and revising the manuscript. All the authors read, revised, and approved the final version of the manuscript.

## Funding

This study was supported by the National Natural Science Foundation of China (Nos: 81971642 and 81771860), the Beijing Natural Science Foundation (No: 7192041), and the National Key Research and Development Plan (No: 2020YFC0122000).

## Conflict of Interest

LL was employed by Sinounion Medical Technology (Beijing) Corporation, Ltd. The remaining authors declare that the research was conducted in the absence of any commercial or financial relationships that could be construed as a potential conflict of interest.

## Publisher's Note

All claims expressed in this article are solely those of the authors and do not necessarily represent those of their affiliated organizations, or those of the publisher, the editors and the reviewers. Any product that may be evaluated in this article, or claim that may be made by its manufacturer, is not guaranteed or endorsed by the publisher.
